# A First Omics Data Integration Approach in Hass Avocados to Evaluate Rootstock–Scion Interactions: From Aerial and Root Plant Growth to Fruit Development

**DOI:** 10.3390/plants13050603

**Published:** 2024-02-22

**Authors:** Gerardo Núñez-Lillo, Excequel Ponce, Clemens P. Beyer, Juan E. Álvaro, Claudio Meneses, Romina Pedreschi

**Affiliations:** 1 Escuela de Agronomía, Facultad de Ciencias Agronómicas y de los Alimentos, Pontificia Universidad Católica de Valparaíso, Quillota 2260000, Chile; gerardo.nunez@pucv.cl (G.N.-L.); excequel.ponce@pucv.cl (E.P.);; 2 Departamento de Fruticultura y Enología, Facultad de Agronomía y Sistemas Naturales, Pontificia Universidad Católica de Chile, Santiago 7820436, Chile; claudio.meneses@uc.cl; 3 Facultad de Ciencias Biológicas, Pontificia Universidad Católica de Chile, Santiago 8331150, Chile; 4 Millennium Nucleus for the Development of Super Adaptable Plants (MN-SAP), Santiago 8370186, Chile; 5 Millennium Institute Center for Genome Regulation (CRG), Santiago 7800003, Chile

**Keywords:** *Persea americana*, plant development, rootstock–scion interaction, sequencing, transcriptomics, metabolomics, mixOmics

## Abstract

Grafting, the careful selection of rootstocks and scions, has played a crucial role maintaining Chilean avocado fruit quality standards in a scenario in which climate change and drought-related issues have considerably decreased avocado fruit production in the last fifteen years. The historical use of seedling rootstocks in Chile has experienced a recent shift towards clonal rootstocks, driven by the potential to produce more consistent and predictable crops. This research aims to compare Hass avocado plants grafted on Mexicola seedling and Dusa^®^ clonal rootstocks in a soilless and protected system using (i) a differential expression analysis of root and leaf samples and (ii) a fruit transcriptomic and metabolomic integration analysis to improve our understanding of rootstock–scion interaction and its impact on avocado tree performance and fruit quality. The results demonstrated that no significant transcriptomic and metabolomic differences were identified at fruit level in the ready-to-eat (RTE) stage for Hass avocado fruit from both rootstocks. However, Hass avocados grafted on the clonal rootstock showed greater aerial growth and slightly increased fruit size than the seedling rootstock due to the enrichment of cell wall-remodeling genes as revealed in leaves and fruit at harvest stage.

## 1. Introduction

Avocado (*Persea americana* Mill.) is an important sub-tropical crop with high demand worldwide, with the dark-skinned cv. Hass being the most commercialized cultivar in the world. Some key quality attributes of the Hass avocado cultivar include skin color, creamy flesh, and the absence of physiological and pathological disorders. Avocado consumption has become prevalent in the human diet due to its high levels of monounsaturated and polyunsaturated fatty acids, particularly its oleic acid content (50–60% total oil content) [1]. This characteristic fatty acid profile, along with its high content of tocopherols, phytosterols, and phenolic compounds, is associated with a reduced risk of cardiovascular diseases and an overall improvement in human metabolism, making avocado consumption a nutritionally and functionally desirable food choice in the human diet [2,3]. 

The Chilean avocado industry has achieved significant economic importance, marked by exponential growth over the past four decades, culminating in the production of 232,202 tons of avocado fruit in 2009. However, climate change and drought-related issues reduced Chilean avocado fruit production to approximately 70% by 2021 [4]. The evolution of novel cultivation strategies to uphold the rigorous quality standards of Chilean avocado fruit production has played a pivotal factor in establishing Chile as an important avocado producer in the global avocado market. 

Extended periods of drought and reduced water availability have a detrimental impact on orchards, influencing tree health, fruit quality, and overall productivity [5]. Research has indicated that avocados may experience reduced fruit size and increased fruit drop in response to low water levels, leading to decreased productivity [6]. A significant agricultural dilemma revolves around the need to boost crops yields while concurrently decreasing water consumption due to escalating water scarcity. In this context, the importance of research and innovation in water-conserving technologies and sustainable farming practices cannot be overstated, playing a crucial role in alleviating the adverse effects of drought and sustainable agriculture emerges as an important strategy in addressing the challenges posed by climate change [7]. In this context, the feasibility of cultivating Hass avocado trees in a soilless system under protected growing conditions has been demonstrated [8]. This approach proved an efficient use of water and fertilizers, fostering prolonged and more continuous tree growth compared to conventional cultivation systems in open field conditions.

Grafting is a prominent and widely adopted approach within the commercial fruit production sector. The careful selection of rootstocks and scions has been of utmost importance optimizing orchard management and enhancing tree performance [9]. Rootstocks facilitate the grafting of preferred scion varieties onto compatible root systems, resulting in heightened disease resistance, adaptability to different environmental conditions, and improved nutrient uptake. These factors significantly influence the nutritional status of avocado trees and exert a considerable impact on tree size and productivity [10,11].

In Chile, most of the avocado rootstocks employed have historically been grown from seeds. Nevertheless, over the past decade, there has been a notable rise in the adoption of clonal rootstocks for avocado cultivation despite their higher cost [8]. This surge can be attributed to the prospect that orchards featuring genetically identical rootstocks hold the potential to be more consistent and predictable crops, more resistant to diseases, show higher productivity, superior fruit quality, and greater adaptability to environmental conditions in comparison to orchards using seedling rootstocks [12]. Considering the benefits provided using the adequate rootstocks and the apparent superiority of clonal varieties, the selection of clonal rootstocks that optimize the use of water, allowing crops to resist the effects of drought, could be a key factor maintaining avocado fruit quality standards.

It is still under discussion whether the supposed benefits of using clonal rootstocks in avocado production are justifiable due to the higher cost associated with their royalties. Research on the performance of different types of rootstocks in avocado cultivation has revealed very controversial results. For instance, it has been observed that avocado trees grafted onto clonal rootstocks, such as Dusa^®^, exhibit a notable increase in the yield of larger and elongated fruits, although there are no discernible differences in postharvest fruit quality [12]. A study comparing seedling and clonal West Indian rootstocks concluded that the non-significant differences identified did not offset the royalty payment associated with the utilization of clonal rootstocks [13]. More recently, an evaluation employing physiological and metabolomic analyses revealed no disparities in fruit quality or postharvest performance between Hass avocado fruits cultivated on Mexicola (seedling) and Duke 7 (clonal) rootstocks [14]. These controversies are probably due to the different agroclimatic and management conditions that greatly affect rootstock performance and avocado tree health and development [15].

Currently, our understanding of the molecular changes that affect Hass avocado trees grafted on different types of rootstocks is limited, and there is a need for research that evaluates the molecular mechanisms that underlie the differences observed between avocado trees cultivated on seedling and clonal rootstocks under controlled production systems as to assess the effect of the rootstock and minimize other confounding effects. Consequently, this study aims to (i) compare plant performance (aerial and roots) of Hass avocados grafted on Mexicola (seedling) and Dusa^®^ (clonal) rootstocks in a soilless and protected system and (ii) evaluate the differences in fruit quality attributes between fruit samples grown on both types of rootstocks. The comparison was conducted using a transcriptomic approach that involves analyzing root and leaf samples at two distinct tree developmental stages (bloom and harvest), while for fruit samples, an integration of transcriptomic and metabolomic approaches was conducted at harvest and at ready-to eat (RTE) stages. The findings from this research aim to enhance our comprehension of the impact of rootstocks on Hass avocado tree performance and fruit quality.

## 2. Results

### 2.1. Developmental Phenotyping of Hass Avocado Plants

**Figure 1:**
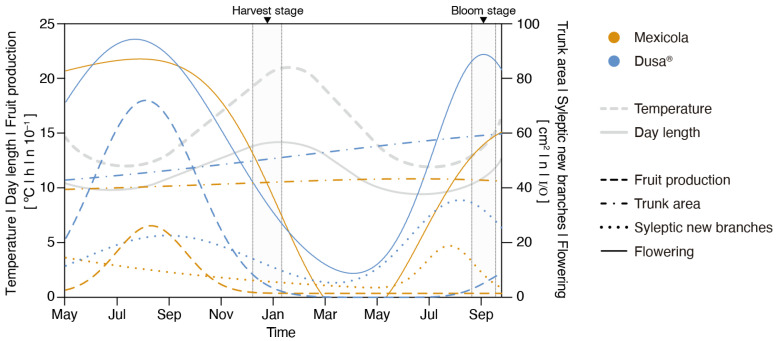
Phenotyping of Hass avocado trees grafted on seedling (Mexicola) and clonal (Dusa^®^) rootstocks during May 2021 and September 2022. Grey highlighted sections correspond to root and leaf sampling periods at full bloom and harvest stages.

The development monitoring of Hass avocados grafted on Mexicola and Dusa^®^ rootstocks under controlled growing conditions is shown in Figure 1. The flowering period (continuous lines) for plants grafted on both types of rootstocks was observed between July and October. Regarding the evaluated parameters of plant growth, the trunk area (dotted–dashed lines) in individuals grafted on Dusa^®^ rootstocks reached 40% of growth between May 2021 and September 2022 (from 43 cm^2^ to 60 cm^2^), twice the observed growth of individuals grafted on Mexicola rootstocks, which only increased 20 % (from 39 cm^2^ to 46 cm^2^). On the other hand, the development of new sylleptic branches was observed between June and September (dotted lines), and a higher number of sylleptic branches were identified in Hass avocados grafted on Dusa^®^ rootstocks. Likewise, in terms of fruit production (Figure 1, dashed lines), it was observed that Hass avocados grafted on Mexicola rootstocks produced fewer fruits than plants grafted on clonal rootstocks. 

Fruit samples harvested from Hass avocados grafted on Mexicola and Dusa^®^ rootstocks were evaluated at harvest and RTE stages (Table 1). Non-significant differences were found in dry matter, firmness and softening rate between fruits grown on the two rootstocks. Regarding fruit size at harvest, the Hass avocado fruit exhibited statistically significant differences in height and width mean values of each group of samples (Table 1, capital letters). Thus, Hass avocado fruits grown on clonal rootstocks showed an average of 10 % increase in height and width compared to fruits grown on Mexicola rootstocks.

### 2.2. Root, Leaf and Fruit Transcriptomic Data Evaluation and Alignment Summary Metrics 

The transcriptomic analysis included two genotypes (Hass avocado trees grafted on Mexicola and Dusa^®^ rootstocks), three tissues (root, leaf and fruit) and two developmental stages for each tissue with three biological replicates. The sequencing produced an average of 56,484,540 reads for each library, with a Q30 value > 94.8 % in each of them. The largest number of sequenced reads was observed for the library M1-RH, with 71,445,590 total reads, and the library with the lowest number of sequenced reads was D1-LB, with 47,999,646 (Table S1). In the sequencing process, no significantly overrepresented libraries were observed. Through filtering out poor-quality reads and searching for adapter sequences, less than 0.7 % of total reads were eliminated for each library from the following analyses. Finally, on average, 85.6 % of total reads were correctly aligned against *Persea americana* var. drymifolia reference genome v3.0 (Table S1). 

### 2.3. Root and Leaf Differential Expression Analysis at Bloom and Harvest Stages 

**Figure 2:**
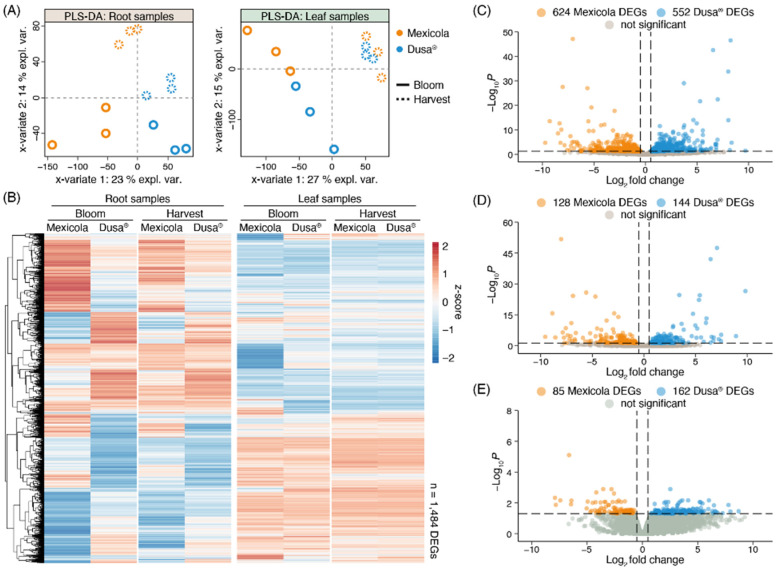
Differential expression analysis between Hass avocado trees grafted on seedling (Mexicola) and clonal (Dusa^®^) rootstocks. (**A**) Partial least square discriminant analysis (PLS-DA) for root (left side) and leaf (right side) samples. (**B**) Red-blue color scale heatmap representing the differentially expressed genes between Mexicola and Dusa^®^ samples at least in one condition. Each column represents the average expression value of three biological replicates scaled considering the mean centered divided by standard deviation (z-score). (**C**,**D**) Volcano plots representing the differentially expressed genes between Mexicola and Dusa^®^ root samples at bloom and harvest stages, respectively. (**E**) Volcano plot representing the differentially expressed genes between Mexicola and Dusa^®^ leaf samples at bloom stage.

Normalized counts of each sequenced library were used to perform the partial least square discriminant analysis (PLS-DA) shown in Figure 2A. For root samples (Figure 2A, left side), it is observed that samples of the different rootstock genotypes (Mexicola and Dusa^®^) were separated with variate 1, while both developmental stages (bloom and harvest) were separated on root samples with variate 2. On the other hand, for samples of avocado leaves cv. Hass grown on the two types of rootstocks (Figure 2A, right side), the bloom and harvest developmental stages were separated with variate 1, and with variate 2 a separation between both types of rootstocks was observed only at the bloom stage. It was also not possible to separate leaf samples grown on both rootstocks at the harvest stage using more variates. Differential expression analyses comparing Mexicola and Dusa^®^ rootstocks were performed for roots and leaves in both developmental stages; in this way, two independent transcriptomic analysis were performed for each tissue, one between Mexicola and Dusa^®^ at the bloom stage, and another between Mexicola and Dusa^®^ at the harvest stage. A total of 1484 differentially expressed genes (DEGs) were identified in at least one condition analyzed, and the expression patterns of each DEG are plotted in the heatmap of Figure 2B. Marked transcriptomic differences can be observed between Mexicola and Dusa^®^ root samples and, as observed in the PLS-DA of Figure 2A, leaf samples show considerably fewer transcriptomic differences only observed at the bloom stage. On the one hand, regarding the differential expression analysis of root samples, as shown in Figure 2C, a total of 1176 DEGs were identified at the bloom stage, while at the harvest stage (Figure 2D), a total of 272 DEGs were identified (approximately 80% fewer DEGs than at the bloom stage). But, when comparing the leaves of avocado trees cv. Hass grafted on both types of rootstocks, 247 DEGs were identified only at the bloom stage (Figure 2E), and no DEGs were identified in the leaf samples at the harvest stage.

### 2.4. Root and Leaf DEG Lists and Gene Ontology Analysis

**Figure 3:**
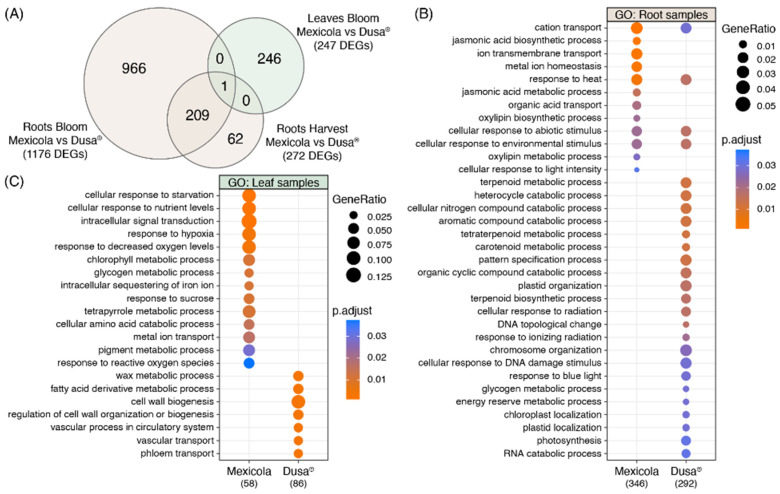
Gene ontology analysis using differentially expressed genes between Hass avocado trees grafted on Mexicola and Dusa^®^ rootstocks. (**A**) Venn diagram comparing the number of DEGs between studied comparisons. (**B**) Gene ontology analysis using the root DEG list. (**C**) Gene ontology analysis using the leaf DEG list.

The number of DEGs obtained in each differential expression analysis shown in Figure 2 is compared in the Venn diagram of Figure 3A. For root samples, 76.8% of DEGs obtained at the harvest stage were shared with those obtained at the bloom stage (209 common DEGs). For this reason, it was decided to use the union of DEGs identified in roots at harvest (1176 DEGs) and bloom (272 DEGs) stages as the total list of root DEGs (1238 DEGs), while for leaf samples, the differentially expressed genes identified at the bloom stage were considered as the total list of leaf DEGs (247 DEGs). Furthermore, only one gene with differential expression in both root and leaf samples was identified, described as a plant invertase/pectin methylesterase inhibitor superfamily protein (INV/PMEi) with approximately 7-fold more expression in roots than in leaves. On the one hand, in roots, greater expression of INV/PMEi was observed in the Mexicola rootstocks both at bloom and harvest stages. In contrast, in leaves, a significant decrease in the expression of INV/PMEi was observed in the Mexicola rootstocks at the bloom stage.

With respect to the list of root DEGs, a gene ontology analysis was performed comparing the DEGs with higher expression values in the Mexicola and Dusa^®^ rootstocks, the results obtained are shown on Figure 3B. In the case of the DEGs with higher expression values in the roots of the Mexicola rootstock, GO terms such as jasmonic acid biosynthetic and metabolic processes, oxylipin biosynthetic and metabolic processes, and metal ion homeostasis stood out, while the GO terms in the group of DEGs with higher expression values in the Dusa^®^ rootstock, the terpenoid biosynthetic and metabolic processes, carotenoid metabolic process, plastid and chromosome organization, glycogen and energy reserve metabolic processes were overrepresented.

On the other hand, in the GO analysis of the leaf DEGs list, it can be observed that the leaves of avocados cv. Hass grafted on Mexicola rootstocks are enriched in GO terms such as cellular response to starvation and nutrient levels, response to hypoxia and decreased oxygen levels, chlorophyll and glycogen metabolic processes, and response to reactive oxygen species, while in the leaves of avocados cv. Hass grafted on Dusa^®^ rootstocks, GO terms such as wax and fatty acid derivative metabolic processes, cell wall biogenesis, and regulation of cell wall organization or biogenesis stood out.

### 2.5. Integration Analysis of Roots’ and Leaves’ Transcriptomic Results

**Figure 4:**
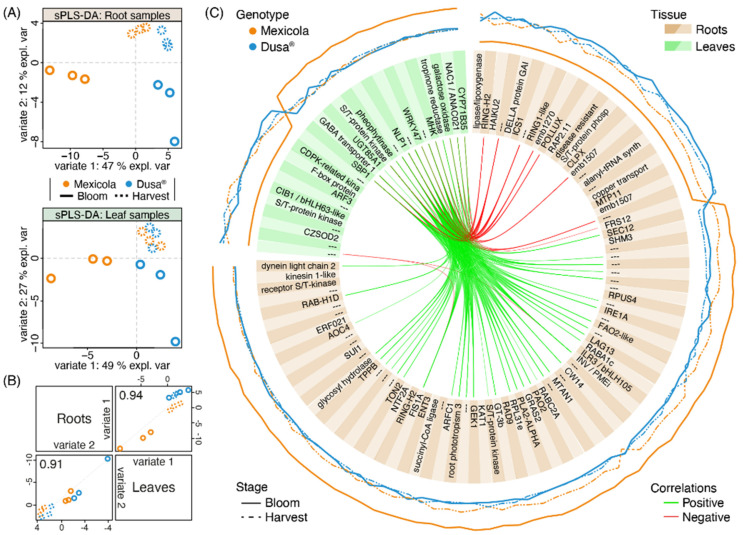
Transcriptomic integration analysis between root and leaf samples of avocado trees cv. Hass grafted on two types of rootstocks, Mexicola (seedling) and Dusa^®^ (clonal). (**A**) Sparse partial least square discriminant analysis (sPLS-DA) with selected variables for root (upper side) and leaf (lower side) transcriptomic datasets. (**B**) Scatterplot from plotDiablo displaying the first (upper diagonal) and second (lower diagonal) sPLS-DA variates with Pearson correlations between root and leaf transcriptomic datasets. (**C**) CircosPlot showing the root and leaf selected transcripts for variate 1. The expression values of each transcript are plotted on the outside part of the circosPlot. Positive and negative correlations between transcriptomic datasets are represented by green and red lines.

To identify transcripts with an association to the observed differences between avocado samples grafted on both types of rootstocks, an integration analysis was carried out using the MixOmics software. The variable selection was carried out to identify the lowest number of transcripts in roots and leaves to maintain the sample distribution observed in the PLS-DA of Figure 2A. In this sense, for roots, 84 and 26 transcripts were selected for variates 1 and variate 2, respectively, while for leaves, 26 and 42 transcripts were selected for variates 1 and variate 2, respectively. The sPLS-DA of roots and leaves using the selected variables is shown in Figure 4A. For both tissues, the selected transcripts allowed us to separate the samples grafted on Mexicola and Dusa^®^ rootstocks at the bloom stage with variate 1, while variate 2 separated both developmental stages (bloom and harvest). The correlation between sample distribution for roots and leaves is shown in Figure 4B and is greater than 0.9 for both variates. Finally, the 110 selected transcripts from roots and leaves associated with variate 1 that discriminate between samples of both types of rootstocks are plotted in the circosPlot of Figure 4C.

Of all these variate 1-selected transcripts, 76 showed high expression values in avocado trees grafted on Mexicola seedling rootstocks, 61 were identified from root tissue (Figure 4C (brown boxes), with the higher expression value represented by the continuous orange line), highlighting a plant invertase/pectin methylesterase inhibitor protein INV/PMEi (the same candidate associated with the common transcripts between leaf and root samples in Figure 3A), the allene oxide cyclase 4 AOC4 (a transcript of the jasmonic acid biosynthetic pathway), and three transcription factors that could be regulating the differences between both rootstocks (GT3b, ILR3/bHLH105 and ERF021). In comparison, the other 15 transcripts were identified in leaf tissue (Figure 4C (green boxes), with the higher expression value represented by the continuous orange line), of which two transcription factors (NAC1 and WRKY47) stood out as overexpressed in Mexicola rootstock-associated plants. On the other hand, of the remaining 34 selected transcripts that showed higher expression values in avocado trees grafted on Dusa^®^ clonal rootstocks, 23 were identified from root tissue (Figure 4C (brown boxes), with the higher expression value represented by the continuous blue line), highlighting an isochorismate synthase ICS1 (a precursor of salicylic acid biosynthesis), a DELLA protein GAI (repressor of gibberellic acid signaling response), and an ethylene-responsive transcription factor RAP2.11. In comparison, from the other 11 transcripts identified in leaf tissue (Figure 4C (green boxes), with the higher expression value represented by the continuous blue line), an F-box protein GID2 (that interacts with DELLA protein and is involved in gibberellic acid signaling) and two transcription factors (ARF3 and CIB1/bHLH63-like) stood out.

### 2.6. Multiomics Integration Analysis of Avocado Fruit Grown on Mexicola and Dusa^®^ Rootstocks

**Figure 5:**
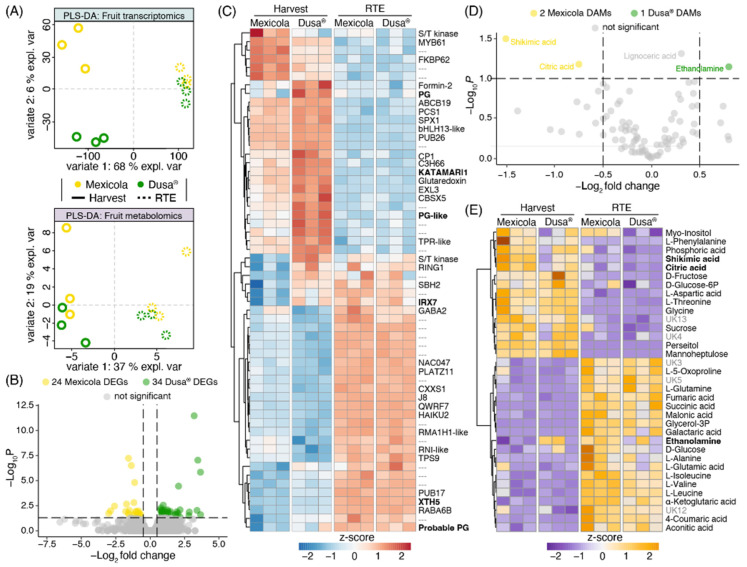
Transcriptomic and metabolomic analysis comparing Hass avocado fruit grown on seedling and clonal rootstocks. (**A**) Partial least square discriminant analysis (PLS-DA) for fruit transcriptomics (upper side) and fruit metabolomics (lower side). (**B**) Differentially expressed genes at the harvest stage are represented in a volcano plot. (**C**) Differentially expressed genes are plotted in a red-blue color scale heatmap. Expression values were scaled considering the mean-centered divided by the standard deviation. (**D**) Differentially abundant metabolites at the harvest stage are represented in a volcano plot. (**E**) Top 35 most significant metabolites are plotted in an orange-purple color scale heatmap. Abundance values were scaled considering the mean-centered divided by the standard deviation.

A transcriptomic and metabolomic partial least square discriminant analysis (PLS-DA) comparing Hass avocado fruit samples from Mexicola and Dusa^®^ rootstocks is shown in Figure 5A. A clear separation was observed with variate 1 between fruit developmental stages (Harvest—RTE) in both datasets. Meanwhile, samples from Mexicola and Dusa^®^ rootstocks were separated at the harvest stage only in the transcriptomic dataset (Figure 5A, upper side) with 6 % explained variance (variate 2), and no separation was observed in the RTE stage. In the same way, no DEGs were found between Hass avocado fruits from seedling and clonal rootstocks at the RTE stage. However, as shown in the volcano plot of Figure 5B, 58 DEGs were identified between groups of avocado samples at the harvest stage, with a *p*-value < 0.05 and |log_2_FC| > 0.5. The red-blue scaled heatmap shown in Figure 5C represents the expression patterns of the 58 DEGs identified at the harvest stage. Notably, two transcription factors, bHLH13-like and C3H66, along with six cell wall remodeling-related genes (three polygalacturonases, one xyloglucan galactosyltransferase, one glucuronoxylan glucuronosyltransferase, and one xyloglucan endotransglucosylase-hydrolase), showed higher expression levels in avocado fruits grown on Dusa^®^ rootstocks. At the same time, the transcription factors MYB domain protein 61 (MYB61) and NAC domain-containing protein 47 (NAC047) exhibited higher expression levels in avocado fruits grown on Mexicola rootstocks.

On the other hand, when comparing the metabolic data of Hass avocado samples grafted on Mexicola and Dusa^®^ rootstocks, no separation of the samples grafted on clonal or seedling rootstocks was observed in any developmental stages analyzed (Figure 5A, lower side) and only three differentially abundant metabolites (DAMs) were identified at the harvest stage with a *p* < 0.05 and |log_2_FC| > 0.5 (Figure 5D). On one side, shikimic acid and citric acid with higher abundance in avocado fruit grown on Mexicola rootstocks were identified. Furthermore, ethanolamine was the only metabolite with more abundance in avocado fruit grown on Dusa^®^ rootstocks. Lignoceric acid also presented significant differences, with higher abundance in samples grafted on Dusa^®^ rootstocks; however, it was discarded for having a log_2_FC = 0.3. No DAMs were identified between avocado fruit grown on seedling and clonal rootstocks at the RTE stage. Finally, the top 35 most informative metabolites (according to an ANOVA test) are plotted in an orange-purple scale heatmap (Figure 5E) with metabolic differences being observed mainly between developmental stages and few differences were found between both rootstocks.

### 2.7. Fruit Multiomics Integration Analysis Using Transcriptomic and Metabolomic Datasets

**Figure 6:**
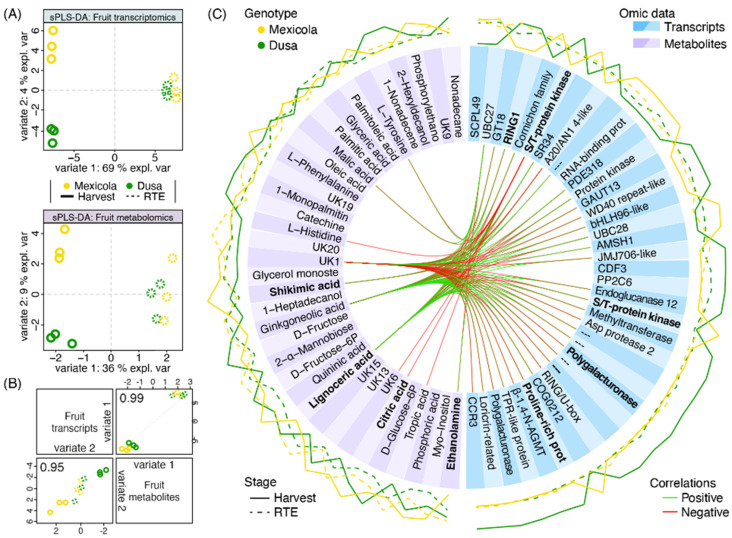
Multiomics integration analysis between Hass avocado fruit grown on seedling and clonal rootstocks. (**A**) Sparse partial least square discriminant analysis (sPLS-DA) with the contribution of transcriptomic (upper side) and metabolomic (lower side) datasets. (**B**) Scatterplot from plotDiablo displaying the first (upper diagonal) and second (lower diagonal) variates with Pearson correlations between omics datasets. Numbers close to 1.0 represent a high correlation between datasets. (**C**) Selected variables obtained with the sPLS-DA are plotted in a circosPlot. Transcriptomic and metabolomic datasets are characterized by blue and purple boxes, respectively. Bold metabolite/gene names represent candidate variables identified by both single- and multiomics analyses.

To integrate transcriptomic and metabolomic information and to find variables that would explain the separation of Hass fruit samples grafted on Mexicola and Dusa^®^ rootstocks, a mixed supervised analysis was carried out with both datasets. First, 17,047 transcripts and 94 metabolites were used to perform a tuning function to choose the optimal number of variables to select for each dataset considering a two-variate analysis. For variate 1, which separates fruit development stages, 100 transcripts and 7 metabolites that best explain the separation between harvest and RTE fruit samples were selected. Regarding the separation between samples grafted on Mexicola and Dusa^®^ rootstocks associated with variate 2, 37 transcripts and 36 metabolites were established. As shown in Figure 6A, a sparse partial least square discriminant analysis (sPLS-DA) with the previously selected variables was built for each dataset. Hass avocado samples grafted on Mexicola and Dusa^®^ rootstocks were separated by variate 2 only at the harvest stage, with 4 and 9% of explained variance for transcriptomic and metabolomic datasets, respectively. High correlations between transcriptomic and metabolomic distribution patterns were found, 0.99 and 0.95 for variates 1 and 2, respectively (Figure 6B). 

Finally, a circosPlot with the correlations between selected transcripts and metabolites for variate 2 was shown in Figure 6C. Through this multiomics analysis, as in the metabolomic analysis shown in Figure 5, the metabolites ethanolamine, shikimic acid, citric acid and lignoceric acid were again selected as candidate metabolites with differential abundance between avocados cv. Hass grown on Mexicola and Dusa^®^ rootstocks. In addition, although they were not statistically significant, it was observed that fatty acids such as palmitic acid, palmitoleic acid and oleic acid presented a tendency to be more abundant in Hass fruit samples from Dusa^®^ rootstock. On the other hand, regarding the transcriptomic data, the presence of genes with a cell wall-remodeling function, such as an endoglucanase and two polygalacturonases, along with two transcription factors (bHLH96-like and CDF3) with higher expression in Hass avocados from Dusa^®^ rootstock, stood out.

## 3. Discussion

Rootstocks play a crucial role in avocado cultivation due to their ability to influence different aspects of plant development such as disease resistance, adaptability to different soil conditions, nutrient absorption, and water use efficiency [10]. But also, rootstocks can be subjected to genetic breeding programs to optimize different traits without the need to genetically alter grafted commercial varieties. However, the greatest effort in genetically improving avocado rootstocks is based on resistance to Phytophthora root rot [16]. In addition, in very heterogeneous crops such as avocados, the use of clonal rootstocks has increased considerably in recent decades because clonal rootstocks offer additional benefits such as genetic uniformity, contributing to the efficiency and overall success of the crop [17]. Many research studies have been carried out comparing the differences between the usage of different types of rootstocks in avocado crops considering disease resistance [18], mineral nutrient uptake [19] or salinity tolerance [20]. However, regarding the comparisons between seedling and clonal rootstocks, the results are largely controversial because different agroclimatic and management conditions greatly affect rootstock performance and avocado tree health and development [12,13,14]. For this reason, a system research model was based on avocado trees grafted on seedling and clonal rootstocks growing in a soilless and protected system (controlled conditions) to avoid the inclusion of confounding effects. Thus, the performance of both rootstocks can be studied without the variability of external environmental and management factors.

To date, it has been reported that Dusa^®^ is a rootstock of medium vigor, size and good compatibility with the Hass scion, mainly chosen for its strong Phytophthora root rot resistance [21], superior fruit quantity [22], and good performance under salt [23] and drought [24] stress. On the other hand, Mexicola rootstock is often chosen for its cold resistance, and our previous studies have shown that it displays less root vigor than Dusa^®^ rootstock, both grafted with Hass cultivar [8]. Regarding the physiological comparison made in this research work between avocado trees grafted on Mexicola and Dusa^®^ rootstocks, it is observable that avocados grafted on the clonal rootstock have greater tree aerial development, in addition to greater fruit production with a slightly significant increase in fruit size than avocado trees grafted on seedling rootstocks. These results differed from those reported by [8], even though similar avocado rootstocks and growing conditions were evaluated. The only difference corresponded to the age of the plants, in our study, trees had a more advanced age.

The differential expression analysis of root and leaf samples showed a tendency to enrich GO terms associated with plant stress in trees grafted on Mexicola rootstock. In contrast, in trees grafted on Dusa^®^ rootstock, more GO terms related to plant growth and cell wall-remodeling functions were observed. On the one hand, in the roots of Mexicola rootstock, the enrichment of genes associated with the oxylipins and jasmonic acid biosynthesis and metabolism processes stands out, widely described as compounds that induce plant defense response against biotic and abiotic stress [25,26]. Dusa^®^ rootstock has been reported to withstand *Phytophtora* attack more effectively [21]; thus, the results indicate that avocado trees grafted on the Dusa^®^ rootstock can develop normally because these rootstocks are able to better resist stress. These differences in the roots probably affect the aerial development of Hass avocados grafted on both rootstocks because no signs of any stress response were observed in the leaves of avocados grown on the Dusa^®^ rootstock. These results suggest that using clonal or seedling rootstocks could differentially regulate plant stress tolerance. Mexicola rootstocks, being more susceptible and activating a greater transcriptional stress response, negatively affect the development of plants grafted on them [27]. These results could explain the differences in tree aerial development of Hass avocados shown in Figure 1.

Regarding the integration of root and leaf transcriptomic data and the subsequent variable selection, a gene described as a plant invertase/pectin methylesterase inhibitor (INV/PMEi) stood out as a candidate gene because (i) it is the only gene with differential expression in both root and leaf tissues (Figure 3A) and (ii) presents higher expression levels in the roots of Mexicola rootstock. The function of this gene family has been described as playing a pivotal role in sucrose metabolism, cellulose biosynthesis, nitrogen uptake, reactive oxygen species scavenging, and osmotic stress adaptation. Still, it also regulates the pectin methyl esterification rate to manage cell adhesion, cell wall porosity, and elasticity [28]. Also, the allene oxide cyclase 4 (AOC4) is a key gene in the jasmonic acid biosynthetic pathway, a hormone widely described in the plant response to biotic and abiotic stress [24]. Considering that according to the GO analysis in Figure 3, the roots of Mexicola rootstock seem to maintain a higher response to stress than Dusa^®^ rootstock, INV/PMEi and ACO4 seem to be excellent candidates to explain the differences in the use of both rootstocks. Several genes with high correlation to INV/PMEi and ACO4 in leaf samples were identified (Figure 4C), among which two transcription factors (NAC1 and WRKY47) were identified. Both candidate transcription factors in leaves have been associated with the plant stress response; the effect of NAC1 has been described in developmental processes, abiotic stress, and pathogen tolerance [29], while WRKY47 was induced in drought stress in rice [30].

Furthermore, the fruit transcriptomic analysis shows that, while fruit are still on the tree, there is an enrichment of genes with cell wall-remodeling function overexpressed in fruit grown on Dusa^®^ rootstock (Figure 5), including three polygalacturonases, one xyloglucan galactosyltransferase, one glucuronoxylan glucuronosyltransferase, and one xyloglucan endo-transglucosylase-hydrolase probably responsible for the slightly increased fruit size obtained for fruit from this rootstock. In the multiomics integration analysis (Figure 6), two polygalacturonases and one endoglucanase were identified as candidates to explain the differences observed between Hass fruit samples from Mexicola and Dusa^®^ rootstocks. Cell wall-related genes have been extensively associated with fruit quality parameters such as softening, fruit size, and development in various fruit species. For instance, the fruit cracking phenotype associated with the softening process has been linked to expression patterns of expansins and polygalacturonases in sweet cherry [31] and tomato [32] cultivars; the expressions of expansin and xyloglucan endotransglycosylase genes have been linked with peach fruit development [33]. The polygalacturonase activity has been positively correlated with softening in banana fruit [34]. In this sense, the slightly larger size of avocado fruit from Dusa^®^ rootstock and the accumulation of genes with cell wall-remodeling functions suggest that this clonal rootstock could provide Hass avocado fruit advantages in terms of fruit growth while they are still on the tree probably because Hass avocado trees grafted on Dusa^®^ rootstock (which seem to be more tolerant to stress) are employing fewer carbon resources in defense mechanisms than avocado trees grafted on Mexicola rootstock, thus allowing slightly greater fruit production and larger fruits.

Six transcription factors differentially expressed at the harvest stage were identified through fruit differential expression analysis (Figure 5) and multiomics integration analysis (Figure 6). Two of them with higher expression in Hass avocado fruit grown on Mexicola rootstocks (MYB61 and NAC047) and four transcription factors with higher expression in Hass avocado fruit grown on Dusa^®^ rootstocks (bHLH13-like, bHLH96-like, C3H66 and CDF3) were identified. Regarding the transcription factors associated with Hass avocado trees grafted on Mexicola rootstock, MYB61 has been related to processes such as mucilage production [35], terpene metabolism and trichome development [36], while for NAC047, although no specific studies have been described, its participation in some gene regulatory networks related to secondary cell wall synthesis [37] and senescence signaling mediated by EIN2 [38] has been reported. Instead, for Dusa^®^ clonal rootstock, the transcription factor bHLH13-like has been described as a negative regulator of jasmonic acid response interacting with JAZ proteins and promoting plant growth [39]. The zinc finger C3H66 has been associated with ABA-mediated regulation, probably associated with ovary/pistil development and fruit set in tomato regulated by NCED1 [40] The transcription factor CDF3 has been reported with multiple functions related to the regulation of flowering time and abiotic stress in *Arabidopsis thaliana* [41], nitrogen use efficiency [42], and enhancing biomass production and yield in tomato [43]. All these transcription factors with high expression values in Hass avocado fruits grown on Dusa^®^ or Mexicola rootstocks associated with fruit development functions could be regulating the gene expression differences of the cell wall-remodeling genes identified and producing the slightly higher fruit sizes observed in Hass avocados from Dusa^®^ rootstock. However, more analyses are still necessary to corroborate this hypothesis. This is the first study, up to our knowledge, to assess under controlled growing conditions, the effect of the rootstock on avocado cv. Hass parameters related to root and aerial plant and fruit levels.

All the differences observed at transcriptomic and metabolomic levels were identified at the harvest stage and disappeared after fruit harvest, suggesting that differentially expressed genes and differentially abundant metabolites are directly associated with the effect of the rootstocks. These results suggest no nutritional or functional differences between Hass avocado fruit grown on Mexicola or Dusa^®^ rootstocks because no transcriptomic or metabolomic differences were observed at the RTE stage. Previous studies have reported no differences at the fruit level and postharvest performance of Hass avocado grown on either seed or clonal rootstocks [14]. These results, together with the results obtained in this work, suggest that the value of clonal rootstocks in terms of nutritional quality and productivity in avocado trees must be interpreted with care and not overstated. However, from the results obtained related to plant growth and fruit until harvest, the apparently slightly better performance of Dusa® rootstock, due to its capacity to cope with stress, should be evaluated under current stressful conditions that affect avocado production (e.g., water stress). This study was conducted in non-limiting conditions related to irrigation and fertigation and in controlled conditions. 

## 4. Materials and Methods

### 4.1. Plant Material

The soilless and protected growing experiments were performed in a greenhouse with natural ventilation in the Pontificia Universidad Católica de Valparaíso (PUCV) facilities in the Province of Quillota, Valparaíso, Chile. The experiment was conducted using a completely randomized block experimental design. Plant material corresponded to avocado trees (*Persea americana*) cv. Hass (5 years after transplanting) grafted on seedling (Mexicola) and clonal (Dusa^®^) propagated rootstocks. Details about tree growth conditions (fertigation, pH, irrigation, drainage) can be found in [8]. Six Hass avocado trees were randomly selected to perform rootstock–scion interaction analysis (three grafted on Mexicola rootstock and three grafted on Dusa^®^ rootstock) at different developmental stages. To analyze different tree developmental stages for root and leaf samples, two important stages related to avocado fruit production and tree performance were selected, the first in September corresponding to the full-bloom stage (flowering period before flower fall begins) and the second in January corresponding to the early harvest stage (development stage focused on fruit growth with less than 20% dry matter). From each of the six selected trees, at both stages, roots (1.0 g of white roots) and leaves (10 completely green leaves) were sampled, frozen and stored at −80 °C. Fruit samples were harvested in March (harvest stage) according to commercial standards which correspond to 23% dry matter content measured digitally using a F-751 Avocado Quality Meter (Felix Instruments, Washington, USA). Fruit size was measured using a Vernier caliper and firmness was monitored from harvest to consumption maturity (RTE stage at 4–8 N of firmness) using a TA.XTplusC Texture Analyser (Stable Micro Systems, Godalming, UK). Mesocarp biopsies from ten fruits for each selected avocado tree (3 biological pooled samples for clonal and 3 biological pooled samples for seedling rootstocks), at both stages, were sampled according to the protocol described by [44], frozen, and stored at −80 °C.

### 4.2. Root, Leaf and Fruit Transcriptomics

Considering that this research involves the analysis of six individuals including three tissues, and for each tissue, two developmental stages, 36 samples were used to perform transcriptomic analysis. Total RNA was extracted from 100 mg of frozen root, leaf and fruit tissues using a Spectrum^TM^ Plant Total RNA kit (Sigma-Aldrich, St. Louis, MO, USA) following the manufacturer’s instructions and stored at −80 °C. Extracted RNA was quantified with a Qubit^®^4.0 fluorometer (Thermo Fischer Scientific, Waltham, MA, USA) using a Qubit^TM^ RNA BR assay kit. RNA integrity was assessed using a Qubit^TM^ RNA IQ assay kit. The IQ number was used to identify RNA integrity with an IQ value over 7.0 to consider good-quality RNA for sequencing.

RNA libraries were constructed according to the TruSeq Stranded mRNA Kit (Illumina, San Diego, CA, USA) following the manufacturer’s instructions. The library concentrations were determined with a Qubit^®^4.0 fluorometer (Thermo Fischer Scientific) using a Qubit^TM^ dsDNA BR assay kit, and library size and integrity were evaluated by capillary electrophoresis using the Fragment Analyzer^TM^ System (Agilent Technologies, Santa Clara, CA, USA) with the DNF-474-0500 HS NGS Fragment Kit. The constructed libraries were sequenced using Macrogen sequencing services (Seoul, Republic of Korea) in paired-end mode on a HiSeqX sequencer.

Raw sequencing data files were evaluated with FASTQC software and read-quality trimming and filtering were conducted using Flexbar v3.5.0 [45]. The STAR aligner software v2.7.10 [46] aligned filtered reads against *Persea americana* var. drymifolia reference genome v3.0. For each library, the *featureCounts* function from the R package Rsubread v2.8.1 [47] was applied to assign expression values to each uniquely aligned fragment. The R package mixOmics v6.20.0 [48] was used to perform a partial least square discriminant analysis (PLS-DA) with the *plsda* function, and scaled expression values are plotted in a color scale heatmap using the R package pheatmap v1.0.12. Differential expression analysis was performed using the R package edgeR v3.36.0 [49] using a trimmed mean of M-values (TMM) normalization method. Differentially expressed genes (DEGs) with a *p* < 0.05 and a |log_2_FC| > 0.5 were plotted in a volcano plot using the R package EnhancedVolcano v1.14.0 (available at https://github.com/kevinblighe/EnhancedVolcano, accessed on 13 December 2023). A gene ontology analysis with the *Arabidopsis thaliana* orthologs of all DEGs was performed with the R package ClusterProfiler v4.0.5 [50] using the *compareCluster* function. The parameters used for this analysis were as follows: the list of variables in each cluster in ENTREZID, *enrichGO* sub-function, the universe from the total of variables that present annotation as genetic background, and a filter of FDR < 0.05. Subsequently, the semantics filter of GO terms was performed using the *simplify* function of the same package using a *p*-value and *q*-value cutoff less than 0.05.

### 4.3. Fruit Metabolite Profiling

Gas chromatography–mass spectrometry (GC-MS) analyses were carried out to achieve the relative quantification of polar and non-polar metabolites. A total of 12 samples only for fruit mesocarp tissues were prepared for metabolomic analysis considering six individuals (three grafted on Mexicola and three grafted on Dusa^®^ rootstocks) and two fruit developmental stages (harvest and RTE). The extraction and derivatization of polar metabolites were carried out by applying the modified protocol described in [51]. Chromatographic peaks were assigned and identified by comparing retention times and mass spectra to a home-built library of commercial standards and the NIST14 library using Mass Hunter Quantitative software (Agilent Technologies). On the other hand, the extraction and derivatization of non-polar metabolites were performed according to the modified protocol described in [52]. The samples were derivatized using 150 µL of *N*-trimethylsilyl-*N*-methyl trifluoroacetamide (MSTFA) and incubated for 30 min at 37 °C with shaking. One microliter of each sample was analyzed through an Agilent 7890B gas chromatograph equipped with a 5977A single-quadrupole mass spectrometer, an electron impact ionization source, a PAL3 autosampler, and a 30 m × 0.25 mm × 0.25 µm DB-5ms column (Agilent Technologies). The injector and interface temperatures were 220 °C and 280 °C, respectively. The helium flow rate was 1 mL min^−1^ and a split ratio of 25:1 was used. The initial oven temperature was 120 °C for 1 min; then, it was increased to 300 °C (5 °C min^−1^) and maintained for 15 min. The source and quadrupole temperatures were 230 °C and 150 °C, respectively. Mass spectra were obtained in the 50 to 600 *m/z* range with a scan rate of 2.66 scans per second. Fatty acids were determined according to the protocol described in [53] with the same chromatographic conditions. Fatty acid methyl esters (FAMEs) were identified and quantified based on retention time comparison with previously injected external standards. The results were expressed as g fatty acid kg^−1^ dry weight (DW). Metabolomic data were scaled considering the mean-centered divided by standard deviation using Metaboanalyst software (available online at https://new.metaboanalyst.ca/MetaboAnalyst/, accessed on 13 December 2023), and data visualization (heatmap, PLS-DA and volcano plot) was performed with the same software.

### 4.4. Multiomics Integration Analysis

Normalized expression/abundance values of transcripts and metabolites were used to perform multiomics integration analysis. The R package mixOmics v6.20.0 [48] was used to perform the multiblock partial least square discriminant analysis (multiblock PLS-DA) considering the representation space of each dataset independently with the *block.plsda* function. Correlations between omics datasets were calculated and plotted with the *plotDiablo* function of the same package. Finally, the *tune.block.splsda* function was used to identify the minimum number of variables that explained the sample dispersion of each variate, and correlations between selected variables were plotted using the circosPlot function considering a correlation cutoff = 0.9.

## 5. Conclusions

The performance of clonal Dusa^®^ and seedling Mexicola rootstocks under controlled growing conditions was realized in this research to determine the impact of rootstock selection on Hass avocado plant development and fruit production. The Dusa^®^ clonal rootstock exhibited slight advantages over Mexicola rootstock, showing greater tree aerial development and slightly increased fruit size. Transcriptomic analyses revealed that Mexicola rootstock exhibited an overexpression of stress-related response genes, while Dusa^®^ rootstock showed an enrichment of plant growth and development-associated genes. The identification of an INV/PMEi and an AOC4, along with the transcription factors NAC1 and WRKY47, provided insights into the molecular mechanisms underlying these differences. Regarding avocado fruit analysis, fruits grown on the evaluated clonal rootstock were slightly larger than those grown on Mexicola rootstock while the fruit was still on the tree, but no significant differences were observed in fruit nutritional or functional aspects at the ready-to-eat stage (consumption stage). Further research under more challenging conditions, such as water stress, is needed to fully assess the performance of clonal and seedling rootstocks in practical avocado farming scenarios. This study contributes valuable insights into the complex interplay between rootstock selection, gene expression, and avocado tree performance, providing a foundation for future research and informed decision making in avocado cultivation practices.

## Figures and Tables

**Table 1:** Fruit phenotyping of Hass avocado samples grafted on Mexicola and Dusa^®^ rootstocks.

Genotype	Sample	Dry Matter (%)	Fruit Size (cm)		Firmness (N)	
			Height	Width	Harvest	RTE
Hass–Mexicola	M1	23.7 ± 1.8 ^a^	7.4 ± 0.5 ^a^	5.1 ± 0.2 ^a^	129.0 ± 26.1 ^a^	10.3 ± 1.7 ^a^
Hass–Mexicola	M2	22.4 ± 1.4 ^ab^	7.7 ± 0.5 ^ab^	5.5 ± 0.3 ^ab^	120.1 ± 9.8 ^a^	08.8 ± 1.3 ^a^
Hass–Mexicola	M3	21.6 ± 1.3 ^b^	8.2 ± 0.5 ^b^	5.9 ± 0.3 ^bc^	125.5 ± 11.8 ^a^	09.5 ± 1.3 ^a^
	**Average**	**22.6 ± 1.7 ^A^**	**7.8 ± 0.6 ^A^**	**5.5 ± 0.4 ^A^**	**125.7 ± 18.1 ^A^**	**09.7 ± 1.5 ^A^**
Hass–Dusa^®^	D1	23.3 ± 1.3 ^ab^	9.0 ± 0.4 ^c^	6.0 ± 0.3 ^cd^	126.5 ± 12.4 ^a^	10.1 ± 2.1 ^a^
Hass–Dusa^®^	D2	22.9 ± 1.8 ^ab^	8.9 ± 0.2 ^c^	6.4 ± 0.2 ^d^	124.9 ± 9.8 ^a^	11.2 ± 1.4 ^a^
Hass–Dusa^®^	D3	22.7 ± 1.2 ^ab^	7.9 ± 0.5 ^ab^	5.8 ± 0.3 ^bc^	130.4 ± 8.5 ^a^	10.6 ± 1.0 ^a^
	**Average**	**23.0 ± 1.4 ^A^**	**8.6 ± 0.6 ^B^**	**6.1 ± 0.3 ^B^**	**127.1 ± 10.4 ^A^**	**10.6 ± 1.7 ^A^**

Lowercase letters represent a one-way ANOVA test between all samples. Capital letters stand for *t*-test statistical differences between Mexicola and Dusa^®^ average values.

## Data Availability

The datasets generated and analyzed for this study can be found in the National Center for Bio-technology Information (NCBI) repository, PRJNA1027005 http://www.ncbi.nlm.nih.gov/bioproject/1027005 (accessed on 11 October 2023).

## Supplementary Materials

The following supporting information can be downloaded at: https://www.mdpi.com/article/10.3390/plants13050603/s1, Table S1: RNA extraction, library construction and sequence processing summary metrics of avocado (*Persea americana* cv. ’Hass’) root, leaf and fruit samples grown on Mexicola and Dusa^®^ rootstocks.
